# Does hypoxia play a role in the development of sarcopenia in humans? Mechanistic insights from the Caudwell Xtreme Everest Expedition

**DOI:** 10.1016/j.redox.2017.05.004

**Published:** 2017-05-08

**Authors:** Liesl Wandrag, Mario Siervo, Heather L. Riley, Maryam Khosravi, Bernadette O. Fernandez, Carl A. Leckstrom, Daniel S. Martin, Kay Mitchell, Denny Z.H. Levett, Hugh E. Montgomery, Monty G. Mythen, Michael A. Stroud, Michael P.W. Grocott, Martin Feelisch, for the Caudwell Xtreme Everest Research Group

**Affiliations:** a Nutrition and Dietetic Research Group, Department of Investigative Medicine, Imperial College London, UK; b Human Nutrition Research Centre, Institute of Cellular Medicine, Newcastle University, Campus for Ageing and Vitality, Newcastle on Tyne NE4 5PL, UK; c Warwick Systems Biology Centre and Warwick Medical School, University of Warwick, Coventry CV4 7AL, UK; d University College London Centre for Altitude Space and Extreme Environment Medicine, UCLH NIHR Biomedical Research Centre, Institute of Sport and Exercise Health, 170 Tottenham Court Road, London W1T 7HA, UK; e Department of Cell and Developmental Biology, Division of Biosciences, University College London, WC1B 6BT, UK; f Clinical & Experimental Sciences, Faculty of Medicine, University of Southampton and University Hospital Southampton NHS Foundation Trust, Southampton SO16 6YD, UK; g Division of Surgery and Interventional Science, University College London, 9th Floor, Royal Free Hospital, London NW3 2QG, UK; h University Hospital Southampton NHS Foundation Trust, Southampton General Hospital, Southampton SO16 6YD, UK; i Southampton NIHR Respiratory Biomedical Research Unit, UK

**Keywords:** Hypobaric hypoxia, Hypoxemia, Lean body mass, Oxidative stress, Nitrite, Inflammation, Interleukin-6, Insulin, Glucagon-like peptide-1

## Abstract

**Objectives:**

Sarcopenia refers to the involuntary loss of skeletal muscle and is a predictor of physical disability/mortality. Its pathogenesis is poorly understood, although roles for altered hypoxic signaling, oxidative stress, adipokines and inflammatory mediators have been suggested. Sarcopenia also occurs upon exposure to the hypoxia of high altitude. Using data from the Caudwell Xtreme Everest expedition we therefore sought to analyze the extent of hypoxia-induced body composition changes and identify putative pathways associated with fat-free mass (FFM) and fat mass (FM) loss.

**Methods:**

After baseline testing in London (75 m), 24 investigators ascended from Kathmandu (1300 m) to Everest base camp (EBC 5300 m) over 13 days. Fourteen investigators climbed above EBC, eight of whom reached the summit (8848 m). Assessments were conducted at baseline, during ascent and after one, six and eight week(s) of arrival at EBC. Changes in body composition (FM, FFM, total body water, intra- and extra-cellular water) were measured by bioelectrical impedance. Biomarkers of nitric oxide and oxidative stress were measured together with adipokines, inflammatory, metabolic and vascular markers.

**Results:**

Participants lost a substantial, but variable, amount of body weight (7.3±4.9 kg by expedition end; p<0.001). A progressive loss of both FM and FFM was observed, and after eight weeks, the proportion of FFM loss was 48% greater than FM loss (p<0.008). Changes in protein carbonyls (p<0.001) were associated with a decline in FM whereas 4-hydroxynonenal (p<0.001) and IL-6 (p<0.001) correlated with FFM loss. GLP-1 (r=−0.45, p<0.001) and nitrite (r=−0.29, p<0.001) concentration changes were associated with FFM loss. In a multivariate model, GLP-1, insulin and nitrite were significant predictors of FFM loss while protein carbonyls were predicted FM loss.

**Conclusions:**

The putative role of GLP-1 and nitrite as mediators of the effects of hypoxia on FFM is an intriguing finding. If confirmed, nutritional and pharmacological interventions targeting these pathways may offer new avenues for prevention and treatment of sarcopenia.

## 1 Introduction

Sarcopenia is defined as a significant loss of fat free mass (FFM). This can affect physical function and lead to an increased risk of physical disability and mortality [1]. The pathogenesis of sarcopenia is likely to be multifactorial, and skeletal muscle hypoxia has been proposed as a causal factor for muscle wasting and reduced contractility [2], [3], [4].

Weight loss has been widely reported in hypoxic chamber experiments and after sojourns at high altitude, with associations between magnitude of loss and both duration of exposure to hypoxia and level of hypoxemia [5], [6], [7]. Current evidence seems to point to an alteration of appetite control and consequent reduction of energy intake as a determinant of the weight loss, whereas energy expenditure (resting and dietary induced thermogenesis) seems to be less affected [8]. More than 60% of the weight lost is typically composed of FFM leading to a decline of muscle contractility and physical performance and, consequently, an individual's capacity to cope with extreme environmental conditions [9].

Changes in appetite hormones [(leptin, ghrelin, glucagon like peptide 1 (GLP-1) and cholecystokinin K (CCK))] have been reported after exposure to hypoxia [10], [11], [12]. In particular, the role of leptin and ghrelin as causal factors in the reduction of energy intake has attracted controversy after studies reported opposing associations of these appetite hormones with weight loss [13], [14], [15], [16], [17]. Recently, a decreased secretion of post-prandial GLP-1 was observed in healthy men exposed to normobaric hypoxia. [18] Conversely, an in vitro study showed enhanced GLP-1 secretion after hypoxia-inducible factor-1α (HIF-1α) knockdown in human adipocytes [19]. To the best of our knowledge, the role of GLP-1 has never been investigated in humans exposed to prolonged hypobaric hypoxia.

A key role in the onset of these adaptive metabolic responses is played by inflammation and oxidative stress, which are enhanced under conditions of low oxygen availability [7], [20], [21], [22]. The consecutive disruption of intracellular redox balance and inflammation both contribute to skeletal muscle mobilization [23], [24]. Hypoxia disrupts the efficiency of the mitochondrial electron transport chain, inducing leakage of electrons to molecular oxygen and thereby generating reactive oxygen species (ROS) [25]. Interleukin 1 (IL-1), interleukin 6 (IL-6) and C-reactive protein (CRP) are often upregulated during expeditions at high altitude [26], and mechanisms linked to skeletal muscle wasting include activation of HIF-1α and NF-κB catabolic pathways and inhibition of the anabolic mammalian target of rapamycin (mTOR) pathway [27], [28], [29]. Nitric oxide (NO) metabolism is also influenced by oxygen availability as demonstrated by raised levels of nitrite and nitrate (markers of NO production) and cyclic guanosine monophosphate (cGMP, a biomarker of NO activity) during acclimatization to high altitude [30].

Therefore, physiological data and plasma samples from the 2007 Caudwell Xtreme Everest (CXE) expedition [31] could provide valuable mechanistic insights into the relationship between hypoxia and FFM loss whereby the extended duration at high altitude serves as a pathogenic model of sarcopenia. We first assess body composition changes (FM and FFM) and fluid shifts [total body water (TBW), intracellular (ICW) and extracellular water (ECW)] that occurred during the expedition. We then evaluate the association between changes in FM and FFM with a comprehensive panel of biomarkers of oxidative stress, inflammation, NO bioavailability, appetite control and intermediary metabolism to identify significant predictors of changes in FM and FFM during prolonged exposure to hypoxia. We hypothesized that changes in FFM would be associated with indices of oxidative stress and inflammation in this group of healthy individuals exposed to hypobaric hypoxia during the CXE study.

## 2 Methods

The study was approved by the University College of London (UCL) Research Ethics Committee, in accordance with the Declaration of Helsinki. Verbal and written informed consent was obtained from all subjects. The study took place between January and June 2007.

### 2.1 Subjects

Twenty-four healthy participants (18 male; mean age 34.9 yr; range 19–59 yr) who were investigators on the CXE 2007 research expedition to Mount Everest participated in this study [31]. All participants were sea level natives free of cardiovascular or respiratory disease. Criteria for participation have been described in detail elsewhere [31].

### 2.2 Study protocol

The design and conduct of this study is described elsewhere [31]. Briefly, participants underwent baseline testing in London (altitude 75 m) before travelling by plane to Kathmandu, Nepal (1300 m). From there they flew to Lukla (2800 m) on expedition day 1 and then trekked to Everest Base Camp (EBC; 5300 m), arriving on expedition day 13. The ascent profile, along with blood hemoglobin levels and oxygen saturations, is detailed in Table S1 of the Online Supplementary Material. Testing was conducted during the ascent in field laboratories at Kathmandu (1300 m; day −3 to 0), Namche (3500 m; day 4–6), Pheriche (4250 m; day 9–10), and at EBC (5300 m; day 15–17, EBC week 1). The participants were divided into two subgroups, Group 1 (base-camp team) who subsequently remained at EBC for the duration of the expedition (n=10) and Group 2 (climbing team; n=14) who subsequently ascended above 5300 m, to a maximum of 8848 m on Mount Everest; measurements were repeated in both groups at week 6 and week 8. Ambient temperatures were well controlled during testing [31].

### 2.3 Dietary intake

Three meals were provided per day along with ample provision of calorific snacks (chocolate, nuts, crisps, malt loaf, cheese, cured meat) ad libitum, guaranteeing access to plenty of carbohydrates and protein to cope with the increased energy cost of the walk /climb; fresh fruit and vegetable intake was low at altitudes above 3500 m. An ample supply of meals and snacks were maintained up to camp 2, beyond this hot food consisted of dehydrated meals whilst snacks remained available.

### 2.4 Peripheral oxygen saturation

SpO_2_ was measured by an independent investigator on the morning of the same day that blood was analyzed, after 10 min of rest, using a pulse oximeter (Onyx 9500, Nonin, USA).

### 2.5 Anthropometry

Body weight (kg) was measured using mechanical Seca 761 scales (Seca, Birmingham, UK), which were hand-calibrated. An independent investigator recorded body weight to the nearest 0.5 kg with the participant wearing base layers only. Height was measured using a portable stadiometer and recorded to the nearest 0.1 cm.

### 2.6 Body composition

Bioelectrical impedance measurements were taken after a 10 min period of supine rest as the participant lay on a non-conducting air mattress with their arms away from their torso and their legs separated. Measurements were obtained before meals and after voiding. The participants wore base layers but removed shoes and socks. Aluminium electrodes were placed on the right side over the metacarpophalangeal joints and between the ulna-radial styloid processes of the wrist and over the metatarsal phalangeal joints and between the malleoli of the ankle. A hand-held, battery operated tetrapolar bioimpedance analyzer (Bodystat®1500) was used to apply currents at multiple frequencies of 5, 50, 100 and 200 kHz. Total body water and extracellular water were calculated by integral algorithms of the bioanalyser. We assumed that water contributed 73% to FFM at normal hydration status. Intracellular water was calculated as the difference between TBW and ECW. FM was calculated as the difference between body weight and FFM.

### 2.7 Blood sampling and sample analysis

All blood samples were taken prior to any exercise protocols scheduled for the day [31] and drawn from the antecubital vein. Plasma was separated from blood cells by centrifugation of whole blood at 800×*g* for 15 min and immediately frozen in 1 ml aliquots in liquid nitrogen. Samples were stored for the duration of the expedition including their transport back to Kathmandu in liquid nitrogen, transported back to the UK on dry ice and kept at −40 °C in a commercial cryostorage facility until analysis. Sample analysis began approximately one year after start of biobanking (July 2007) and was completed within 22 months (Aug 2008-May 2010).

### 2.8 Oxidative stress markers

Isoprostanes (8-iso-prostaglandin F-2α) and 4-hydroxy-2-nonenal (4-HNE) were quantified using direct competitive enzyme immunoassay (Assay Designs, Ann Arbor, MI) and ELISA (OxiSelect HNE-His Adduct ELISA kit, Cell Biolabs, San Diego, CA), respectively. Protein carbonyls (ProCO) were quantified following derivatization with dinitrophenylhydrazine using a commercial ELISA kit (OxiSelect STA-310; Cell Biolabs).

### 2.9 Nitric oxide biomarkers

NO metabolite concentrations were quantified immediately after thawing of frozen plasma aliquots in the presence of an excess of N-ethylmaleimide (NEM, in PBS; 10 mM final concentration). For nitrite/nitrate analysis, NEM-treated samples were deproteinized with ice-cold methanol (1:1 v/v), cleared by centrifugation and subjected to analysis by high pressure liquid chromatography using a dedicated nitrite/nitrate analyzer (ENO20, Eicom). Cyclic GMP was determined by commercial immunoassay kits (R&D Systems, Abingdon, UK; Biomedica, Vienna, Austria).

### 2.10 Inflammatory markers

Interleukin-6, tumour necrosis factor (TNF-α) and macrophage migration inhibitory factor (MIF) were quantified using xMAP technology (Human-Cytokines-12-plex-panel, BioRad) on a Luminex/Bio-Plex-200 System with high-throughput fluidics (BioRad).

### 2.11 Glucose and insulin

Plasma glucose levels were measured with the glucose oxidase method. Insulin levels were determined using the Bio-Plex-Pro-Human-Diabetes 12-plex panel (BioRad).

### 2.12 Metabolic hormones and adipokines

Adiponectin, adipsin, ghrelin, glucose-dependent insulinotropic polypeptide (GIP), GLP-1, glucagon, leptin, resistin, and visfatin were measured with the Bio-Plex Pro-Human-Diabetes multiplex immunoassay panels (BioRad). Epinephrine and Norepinephrine were quantified using a direct competitive enzyme immunoassay (Bi-CAT ELISA, Alpco Diagnostics, Salem, NH). Lactate concentrations were determined colorimetrically by coupled enzymatic reactions using commercial assay kits (Sigma). Triiodothyronine (T3) was measured using the rat thyroid hormone panel Milliplex MAP kit (Millipore; there is no difference between rat and human T3).

### 2.13 Statistical analysis

Data were described as means±SD. Data were log-transformed if not normally distributed (ΔSpO_2_, Δ4-HNE). T test for independent samples were used to compare characteristics between groups 1 and 2 at baseline and during the expedition. Linear mixed models for repeated measures were used to analyze whether there were significant changes in body composition during the expedition. Paired *t*-test was used to test within-subject differences of FM and FFM and ECW and ICW at each phase. Pearson's correlation was used to evaluate significant associations between changes in body composition with metabolic, vascular and oxy-inflammatory biomarkers. For the correlation analysis, we applied the Bonferroni correction to account for the multiple comparisons, and only results with p<0.001 were considered as statistically significant. Stepwise multiple linear regression was performed to identify significant metabolic predictors explaining the loss of FFM during the CXE expedition. Regression models include changes for significant biomarkers identified by the correlation analyzes and measured during the different phases of the expedition (n=138). Analyzes were conducted using SPSS-19 for Windows and Sigmaplot-11 for Windows. P value was set at <0.05.

## 3 Results

### 3.1 Baseline characteristics

Twenty-four participants (14 climbing team and 10 base-camp team) completed the eight-week expedition. Participants were predominantly male (18/5), with mean age and BMI of 34.9±9.2 years and 24.9±2.8 kg/m^2^. Baseline body composition characteristics were not significantly different between participants reaching the summit and those residing at basecamp (Table 1).

**Table 1:** Body composition characteristics of the CXE 2007 Expedition Team and comparison between team members climbing above Everest Base Camp/reaching the summit (climbers) and members residing at Everest Base Camp at the beginning of the expedition.

	**All**	**Climbers**	**Base Camp**	**p**
**N**	24	14	10	–
**Gender (M/F)**	18/6	12/2	6/4	–
**Age, years**	34.9±9.2	35.7±6.8	33.8±12.1	0.62
**Weight, kg**	77.6±12.4	80.7±13.3	72.2±9.5	0.08
**Height, cm**	176.2±6.8	178.4±5.9	172.7±7.2	0.07
**Body Mass Index, kg/m**^**2**^	24.9±2.8	25.2±3.2	24.1±2.2	0.80
**Fat mass, kg**	14.1±5.1	14.8±5.6	13.2±4.5	0.45
**Fat free mass, kg**	63.4±10.1	66.5±9.3	59.2±10.1	0.08
**Total body water, L**	45.4±7.1	47.4±6.4	42.6±7.5	0.10
**Extracellular water, L**	19.1±2.3	19.8±2.2	18.1±2.3	0.08
**Intracellular water, L**	26.0±4.6	27.2±1.1	24.2±4.8	0.12

Data are presented as means±SD. N=number of subjects; M=Male; F=Female; Independent *t* test was used to determine differences between the two groups.

### 3.2 Body composition

**Fig. 1:**
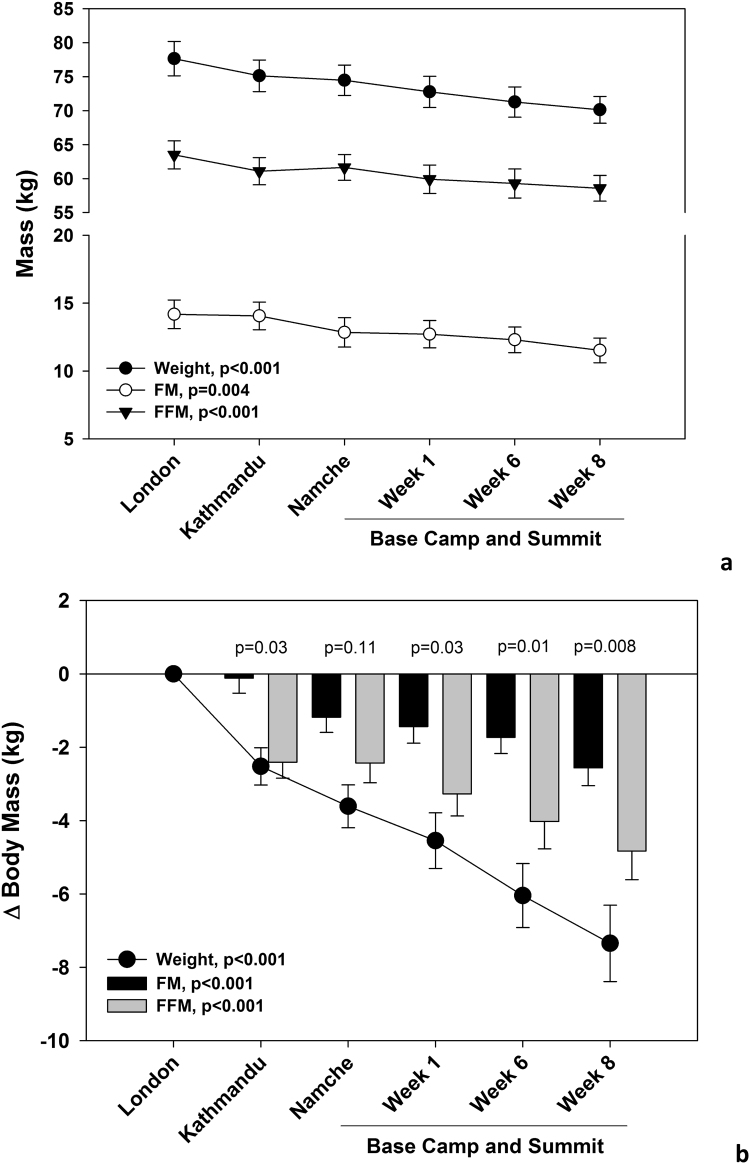
Overall participant mean changes (±s.e) in body weight, fat mass (FM) and fat free mass (FFM) during the expedition. Data are presented are absolute values (Fig. 1a) and as changes (Δ) relative to baseline measured at sea level (London, Fig. 1b). Linear mixed models for repeated measures were used to analyze whether there were significant changes in body composition during the expedition for both sets of data. Paired *t*-test was used to test for differences in losses of FM and FFM at each phase (Fig. 1b).

**Fig. 2:**
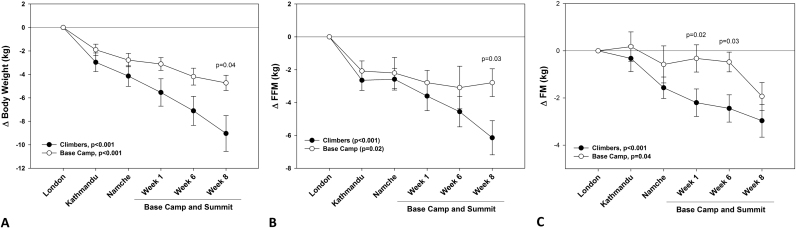
Mean changes (±s.e) in body weight (A), fat free mass (FFM, B) and fat mass (FM, C) during the expedition in individuals attempting to/reaching the summit (climbers) or residing at Everest Base Camp. Data were expressed relative to sea level (London). Linear mixed models for repeated measures were used to analyze whether there were significant changes in body composition during the expedition. Independent *t*-test was used to test for differences in losses of weight, FM and FFM between the two groups at each phase.

Participants lost a significant amount of weight, FM and FFM throughout the expedition (Fig. 1A). Body weight change amounted to −7.3±4.9 kg (p<0.001) at the end of the expedition (Fig. 1B), which was significantly higher in the climbing team compared to base-camp team (−4.3±9.1 kg, p=0.04, Fig. 2A). Body weight changes showed remarkable inter-individual variability, with some participants losing >10 kg and others gaining a little in the climbers whereas variability of weight change was lower for the base-camp team (see Fig. S1 of the Online Supplementary Material for details). Overall, participants showed a progressive loss of both FM and FFM, but the proportion of FFM loss was 48% greater than FM loss (p<0.008) (Fig. 1B). After eight weeks, the climbing team showed a greater loss of FFM (p=0.03, Fig. 2B), whereas a significant difference in FM loss between the two groups was found after one (p=0.02) and six (p=0.03) weeks of the expedition (Fig. 2C).

**Fig. 3:**
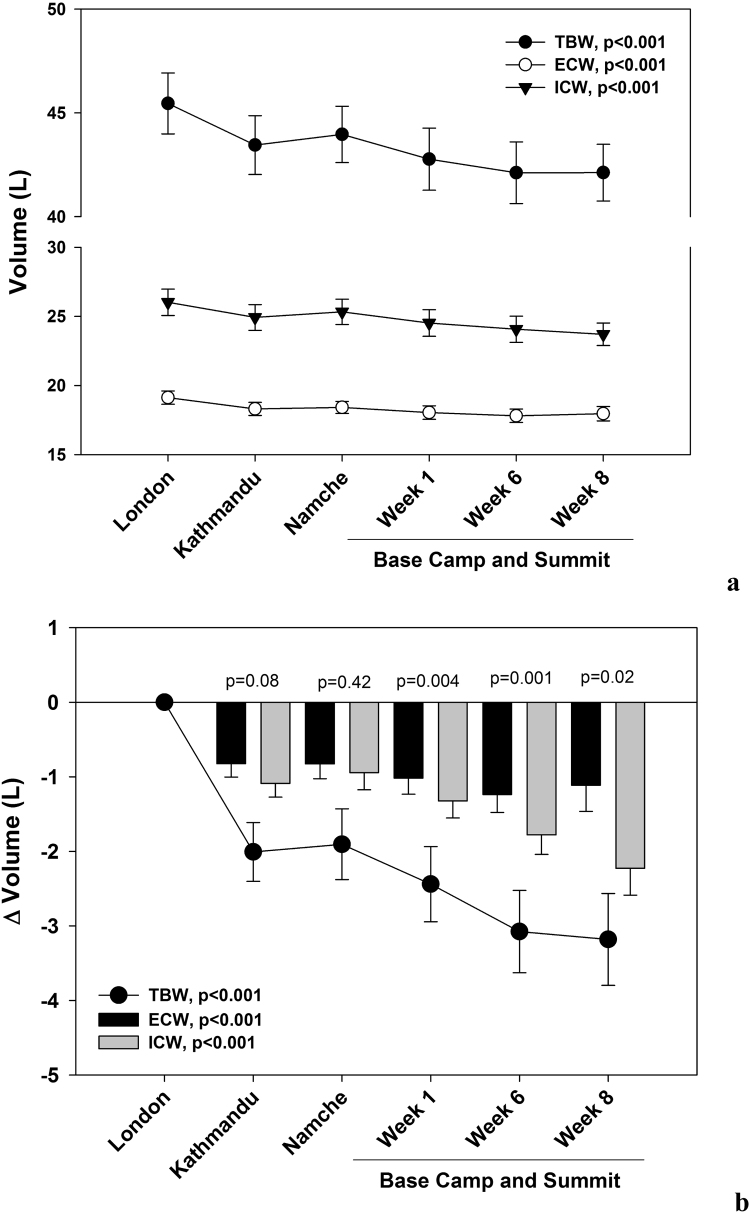
Mean changes (±s.e)in total body water (TBW), extracellular (ECW) and intracellular (ICW) water during the expedition. Data are presented as absolute values (Fig. 3a) and as changes in volume (Δ) relative to baseline measured at sea level (London, Fig. 3b). Linear mixed models for repeated measures were used to analyze whether there were significant changes in body composition during the expedition for both sets of data. Paired *t*-test was used to test for differences in losses of ECW and ICW at each phase (Fig. 3b).

Participants also experienced a significant decline in TBW, ECW and ICW (Fig. 3A). Changes in TBW were −3.1±2.9 L, which accounted for >60% of FFM losses. The majority of TBW losses were accounted for by changes in ICW (−2.2±1.7 L), which were significantly (p=0.02) greater than ECW (−1.1±1.6 L) after eight weeks (Fig. 3B).

### 3.3 Metabolic, vascular and oxy-inflammatory biomarkers

With one exception, all plasma biomarkers showed significant changes during the expedition (p<0.001); only ghrelin concentrations remained unchanged (p=0.54). Nitrite levels increased during the expedition, but a substantial drop was observed at eight weeks at high altitude. Similarly, after an initial rapid increase during the ascent, cGMP concentrations progressively declined during the remainder of the expedition. Changes were more distinct for nitrate, which showed levels below baseline in the last two weeks. ProCO (decline), 4-HNE (increase), 8-isoPGF (increase), IL-6 (increase) showed significant changes with prolonged exposure to hypoxia whereas minor changes were seen for CRP and TNF-α. Increases in concentration during the last two weeks of the expedition were seen with GLP-1, insulin, T3, adrenalin, glucagon, lactate, adiponectin, GIP, adipsin and visfatin. Conversely, a decline was observed for noradrenalin, leptin and resistin (see Fig. S2 of the Online Supplementary Material for details).

### 3.4 Correlation analyzes

We evaluated the association between changes in body composition (weight, FFM and FM) with biomarkers of NO bioavailability, oxy-inflammatory stress, metabolic status and adipokines, and these results are detailed in Table 2. Lower SpO_2_ was associated with a greater decrease in weight (r=0.49, p<0.001), FFM (r=0.38, p<0.001) and FM (r=0.32, p<0.001). Changes in nitrite concentrations were associated with significant changes in FFM (r=−0.29, p<0.001) but not with FM (r=−0.03, p=0.68). A decrease in ProCO was associated with greater losses of body weight (r=0.55, p<0.001), FFM (r=0.42, p<0.001) and FM (r=0.40, p<0.001). Conversely, weight (r=−0.32, p<0.001) and FFM (r=−0.29, p<0.001) losses were associated with an increase in 4-HNE concentrations. The only inflammatory biomarker associated with body composition changes was IL-6 (BW, r=−0.47; FM, r=−0.29; FFM, r=−0.38; p<0.001). Changes in FM were associated with adrenalin (r=−0.32, p<0.001) and lactate (r=−0.29, p<0.001) whereas changes in FFM were associated with GLP-1 (r=−0.47, p<0.001), insulin (r=−0.36, p<0.001), glucagon (r=−0.44, p<0.001) and visfatin (r=−0.41, p<0.001).

**Table 2:** Correlation between changes (Δ) in body components with changes in biomarkers of oxygen exposure and biomarkers of nitric oxide (NO) activity, oxidative stress, inflammation and metabolism (N=138).

		Δ BW	Δ FM	Δ FFM
Hypoxia	Δ SPO_2_	**0.49 (p<0.001)**	**0.32 (p<0.001)**	**0.38 (p<0.001)**

NO Pathway	Δ NO_2_^−^	**−0.30 (p<0.001)**	−0.09 (p=0.27)	**−0.29 (p<0.001)**
	Δ NO_3_^−^	−0.09 (p=0.26)	0.004 (p=0.96)	0.13 (p=0.11)
	Δ cGMP	−0.04 (p=0.58)	−0.03 (p=0.68)	−0.04 (p=0.61)

Oxidative Stress	Δ ProCO	**0.55 (p<0.001)**	**0.40 (p<0.001)**	**0.42 (p<0.001)**
	Δ 4-HNE	**−0.32 (p<0.001)**	−0.22 (p=0.009)	**−0.29 (p<0.001)**
	Δ isoPGF	−0.12 (p=0.15)	−0.10 (p=0.25)	−0.10 (p=0.26)

Inflammation	Δ IL-6	**−0.47(p<0.001)**	**−0.29 (p<0.001)**	**−0.38 (p<0.001)**
	Δ CRP	−0.03 (p=0.71)	−0.06 (p=0.44)	−0.03 (p=0.72)
	Δ TNF-α	−0.06 (p=0.49)	0.13 (p=0.13)	−0.16 (p=0.07)

Metabolic	Δ GIP	−0.14 (p=0.08)	0.11 (p=0.17)	−0.21 (p=0.01)
	Δ Ghrelin	**−0.33 (p<0.001)**	−0.24 (p=0.004)	−0.26 (p=0.002)
	Δ GLP-1	**−0.53 (p<0.001)**	−0.27 (p=0.003)	**−0.47 (p<0.001)**
	Δ Insulin	**−0.44 (p<0.001)**	−0.27 (p=0.003)	**−0.36 (p<0.001)**
	Δ T3	−0.10 (p=0.24)	−0.18 (p=0.02)	−0.005 (p=0.95)
	Δ Adrenalin	**−0.29 (p<0.001)**	**−0.32 (p<0.001)**	−0.17 (p=0.05)
	Δ Nor-adrenalin	0.20 (p=0.02)	0.10 (0.26)	0.22 (p=0.01)
	Δ Glucagon	**−0.48 (p<0.001)**	−0.21 (0.01)	**−0.44 (p<0.001)**
	Δ Lactate	−0.22 (p=0.01)	**−0.29 (p<0.001)**	−0.10 (p=0.22)

Adipokines	Δ Leptin	0.14 (p=0.09)	0.02 (p=0.74)	0.17 (p=0.04)
	Δ Resistin	−0.15 (p=0.07)	−0.02 (p=0.80)	−0.15 (p=0.07)
	Δ Visfatin	**−0.45 (p<0.001)**	−0.23 (p=0.02)	**−0.41 (p<0.001)**
	Δ Adiponectin	−0.04 (p=0.57)	0.06 (p=0.46)	−0.09 (p=0.24)
	Δ Adipsin	−0.08 (p=0.31)	−0.06 (p=0.44)	−0.10 (p=0.23)

Coefficients of correlations r are shown (p value). We applied the Bonferroni correction to the correlation analysis to account for multiple comparisons, and only results with p<0.001 were considered as statistically significant (highlighted in bold). BW: body weight; FM: fat mass; FFM: fat free mass; SPO_2_: oxygen saturation; NO_2_^−^: nitrite; NO_3_^−^: nitrate; cGMP: cyclic guanylate monophosphate; ProCO: protein carbonyls; 4-HNE: 4-hydroxynonenal; isoPGF: 8-iso-15(S)-Prostaglandin F_2α_; IL-6: interleukin 6; CRP: C-reactive protein; TNF-α: tumour necrosis factor α; GIP: gastric inhibitory polypeptide; GLP-1: glucagon-like peptide 1; T3: triiodothyronine.

### 3.5 Stepwise multiple regression

FFM losses were predictive of changes in GLP-1 (B±SE, −0.006±0.001, p<0.001), insulin (B±SE, 0.002±0.001, p=0.01) and nitrite (B±SE, −4.7±2.2, p=0.03) in a fully adjusted multivariate model. Changes in ProCO (B±SE, 0.16±0.07, p=0.03) were instead the only predictor of FM changes (Table 3).

**Table 3:** Stepwise multiple linear regression to identify significant metabolic predictors explaining the loss of FFM and FM during the Caudwell Xtreme Everest Expedition.

	B	SE	P
**Dependent Variable:** ΔFFM (R^2^=0.34)			
Constant	−1.47	0.31	<0.001
ΔGLP−1	−0.006	0.001	<0.001
ΔInsulin	0.002	0.001	0.01
ΔNO_2_^−^	−4.7	2.2	0.03

**Dependent Variable:** ΔFM (R^2^=0.23)			
ΔProCO	0.16	0.07	0.03

Regression models include changes for significant biomarkers identified by the correlation analyzes and measured across the different study expedition phases (N=138). Dependent variable is change in FFM. Independent variables in the FFM model were changes in SPO_2_, nitrite (NO_2_^−^), cyclic guanosine mono phosphate (cGMP), protein carbonyls (ProCO), interleukin 6 (IL-6), glucagon, visfatin, 4-hydroxynonenal (4-HNE) and glucagon-like peptide 1 (GLP-1). Independent variables in the FM model were changes in SPO_2_, protein carbonyl (ProCO), interleukin 6 (IL-6), adrenalin and lactate.

## 4 Discussion

### 4.1 Summary of main findings

Our analyzes explored whether data from healthy humans exposed to hypobaric hypoxia at high altitude could provide mechanistic clues into the pathogenesis of sarcopenia and identify potential physiological targets for intervention to preserve FFM in humans. First, we demonstrated a significant direct association between lower oxygen saturations and loss of FM and FFM. The correlation analyzes identified two distinct groups of biomarkers: 1) *compensatory* – loss of FM and FFM was associated with increased biomarker concentrations; and 2) *synergistic* – loss of FM and FFM was associated with decreased biomarker concentrations. The former includes nitrite, 4-HNE, IL-6, GLP-1, insulin, adrenalin, glucagon and visfatin, whereas the only biomarker to show synergistic changes with FM and FFM was ProCO. The multivariate analyzes provide further support for the role of GLP-1, nitrite and insulin as mediators of FFM loss, whereas the only predictor of FM loss were changes in ProCO concentrations.

### 4.2 Body composition

Data from previous studies have shown that the extent and rate of weight loss is correlated with the rate, extent and duration of the exposure to hypoxia [5], [6], [32], [33], [34], but the factors involved in the onset and maintenance of this remain undetermined. Variability in the magnitude of weight loss in the face of a consistent exposure (rate, extent and duration) to hypobaric hypoxia in our participants is striking and consistent with previous reports [5], [6], [7], [34]. This variability in response to a consistent stimulus provided the signal on which our analysis is based. Since rate, extent and duration of the hypoxic stress experienced during this expedition were virtually identical for all members of the climbing and laboratory team the variability in response between the two groups must therefore be due to either genetic or epigenetic differences in metabolic phenotype. Differences in FM loss were particularly striking (Fig. 2C) inviting speculation about differential fatty acid handling, regulation of fat synthesis and lipolysis or a combination of these factors between individuals with different levels of prior high altitude experience (criteria for inclusion in the climbing team included previous event-free ascents above 6500 m and over 8000 m for summit climbers, whereas members of the laboratory staff must have had event-free exposure to altitudes >4000 m [31]). Since those differences appear to resurface upon a subsequent hypoxic challenge already at moderate altitudes (i.e. during the early phase of this study before separation of the Core Team into EBC laboratory staff and climbers) and are therefore related to the process of acclimatization to hypoxia this aspect of metabolic regulation would seem to merit further study.

Processes regulating appetite and energy intake along with hypoxia-induced suppression of skeletal muscle protein synthesis seem to have a more prominent role than energy expenditure per se in driving weight loss [8]. We could not directly test the specific contribution of these two components to the individual changes in energy balance during the expedition, but the significant association of satiety hormones (insulin, GLP-1) with FFM loss and the greater body composition changes in climbers may indicate that both sides of the energy balance equation (lower energy intake and increased physical activity/energy expenditure, in particular in the climbing group) could be involved. Alternatively, hypoxia may directly suppress protein synthesis via the mTOR/Akt pathways. However, none of these factors on their own can possibly account for the observed differences in weight loss between laboratory and climbing team (Fig. 2) as both groups adhered to the exact same ascent profile until reaching EBC.

Weight losses of 2.4–5.0 kg have been reported during expeditions to altitudes above 5000 m (duration: 14–47 days) [5], [7], [33], [35]. The use of hypoxic chambers, where food intake, temperature and exercise were controlled in two independent studies, resulted in average weight loss ranging from 5.0 to 7.4 kg in subjects exposed to hypobaric hypoxia (simulated altitude of 8848 m) over 38 days [32], [36], which are comparable to the mean weight loss observed in our cohort.

Our study showed that the FFM loss contributed to more than 50% of total weight lost, which clearly deviates from the Forbes's theory which states that approximately one-fourth of weight loss will be FFM (i.e., ΔFFM/ΔWeight=~0.25) with the remaining three-fourths fat mass [37], [38]. The average water content of FFM is about 73% and therefore it is not surprising that TBW accounted for the majority of FFM losses [39]; however, the higher loss of ICW compared to ECW indicates a highly catabolic state and mobilization of metabolically active body cell mass [40]. These changes in body composition have been a common observation in studies conducted at high altitude and it could represent an adaptive protective mechanism under conditions of reduced oxygen availability [9]. Murray & Montgomery have advanced the hypothesis that free amino acids and ketone bodies may act as both substrates and metabolic modulators to protect cells from the hypoxic challenge by improving mitochondrial efficiency, activating ATP-sensitive potassium channel and reducing the production of reactive oxygen species [41]. The Operation Everest II chamber study reported a 2.5 kg loss of FM and a 4.9 kg loss of FFM [32] which are similar to the changes reported in our study. Despite the significant difference in physical exertion, this similarity in body composition changes would seem to provide further support for the role of hypoxia as a key factor in the pathogenesis of sarcopenia.

### 4.3 Oxidative stress and inflammation

Oxidative stress, chronic inflammation, and mitochondrial dysfunction all play important roles in muscle atrophy [42]. The interaction of these factors may converge on several intracellular signaling pathways, which affect the balance between protein synthesis and breakdown, and induce apoptosis, underpinning the primary pathology of significant muscle mass loss [43]. This model implies a predominant, damaging role of ROS on skeletal muscle, but the physiological signaling roles of ROS and NO generated during muscle contractile activity cannot be dismissed [44]. In skeletal muscle, NAD(P)H oxidases and nitric oxide synthases are the main producers of superoxide (O_2_^−^) and NO, respectively, which may interact with each other to form the potent oxidant, peroxynitrite (ONOO^−^) [45]. However, the ROS that seems to play a major signaling role in the muscle is hydrogen peroxide (H_2_O_2_) [44], [46]. These reactive species are characterised by different chemical properties which may activate a number of transcription factors (NF-kB, AP-1, HSF-1 and Nrf2), modify protein activities and potentially induce downstream redox effects such as stimulation of cytoprotective and metabolic mechanisms such as autophagy, muscle repair and mitochondrial biogenesis [44], [45], [46].

The univariate correlations show a significant association between loss of FFM and plasma 4-HNE concentrations, suggesting the involvement of oxidative stress (contributing to lipid oxidation) in muscle mobilization. ProCO was the only significant predictor of FM loss in the multivariate model. Plasma levels of ProCO are a measure of protein oxidation promoted by ROS but, counter intuitively, the concentrations of ProCO progressively decreased with FM mobilization. This might be explained by a predominant utilisation under these conditions of proteins as a metabolic fuel; alternatively, skeletal muscle turnover may be enhanced to supply specific amino acids for which there is a higher demand in hypoxia [41]. Another reason for the relatively greater muscle loss in the context of the elevated inflammatory response may be that the amino acid content of acute phase proteins is very different to that of normally produced transport and structural proteins (and indeed to that provided in most diets) [47]. Acute phase proteins have a particularly high phenylalanine, tyrosine and tryptophan content; for example, the phenylalanine content of acute-phase proteins is 105 g/kg versus 40 g/kg in muscle, meaning that to build 100 g of acute-phase protein one would need about 100×105/40 g of protein substrate from food or catabolism.

IL-6 concentrations were significantly elevated, and this was the only pro-inflammatory marker associated with changes in FFM and FM. The physiological role of IL-6 as either a pro-inflammatory cytokine or adaptive myokine is still controversial [48], and our study was not suited to distinguish between these roles. However, we speculate that these two physiological functions may not be mutually exclusive but rather be part of the same adaptive process; the inflammatory response could be part of an immune, protective change induced by the hypoxic stress whereas the myokine metabolic action could be involved in the enhancement of muscular efficiency as part of the response to increased energetic demands of the remaining muscle fibres, aimed at compensating FFM loss.

A short exposure to a 4200 m simulated altitude increased IL-6 concentrations with and without exercise [49]. In 48 trekkers studied over a 6-week ascent up to 5129 m, IL-6 and IL-17a levels were found to be significantly higher at high altitude than at sea level, and exercise led to a further significant increase in IL-6 but not IL-17 levels [50]. While IL-6 seems to be critically involved in hypoxia-induced adaptive changes in muscle metabolism [51] it remains to be established whether these responses have also a defined role in the pathogenesis of sarcopenia.

### 4.4 Nitric oxide

Nitrite and cGMP concentrations progressively increased during the ascent and showed a slight decline after eight weeks, but concentrations did not drop below baseline. Changes in nitrate concentrations were more defined and a substantial decrease compared to baseline was observed after six weeks. Levett et al. [30] have previously explored part of these responses and interpreted them as being indicative of an enhanced conversion of nitrate into nitrite and NO as a consequence of prolonged hypoxic exposure. The novel finding of the current analysis is the significant association between FFM loss and circulating nitrite concentrations. Again, this could be part of an orchestrated compensatory response to enhance muscular energy efficiency under conditions of low oxygen availability and reduced muscle mass. This hypothesis is supported by the role of NO in mitochondrial biogenesis and efficiency [52] as demonstrated by the enhanced Phosphate/Oxygen Ratio in skeletal muscle after supplementation with dietary nitrate [53]. In addition, inorganic nitrate supplementation in participants exposed to normobaric hypoxia increases exercise performance and reduces oxygen consumption, effects which were significantly correlated with post-supplementation plasma nitrite concentrations [54]. Of note, skeletal muscle has recently been proposed to act as endogenous nitrate reservoir; thus, the increase in circulating nitrite may be secondary to the reduction of released nitrate [55].

### 4.5 Metabolic responses

GLP-1 is an incretin hormone secreted by L-cells in the small intestine in response to eating and is involved in the regulation of glucose levels, gastric emptying and appetite [56]. GLP-1 also appears to cause insulin-independent vasodilation and potently stimulates NO synthase phosphorylation in endothelial cells [57]. Moreover, GLP-1 recruits muscle microvasculature, which is associated with increased muscle glucose utilisation, plasma NO metabolite concentrations, muscle interstitial oxygenation as well as insulin clearance and uptake [58]. Our results clearly highlight the pleiotropic effects of hypoxia on multiple metabolic pathways. FM mobilization was associated with increased concentrations of counter-regulatory hormones such as adrenalin and lactate as they are linked to activation of β-oxidation and anaerobic metabolism. In a previous analysis, we demonstrated a significant association between adrenalin and insulin sensitivity [20], suggesting an activation of lipolytic pathways to satisfy enhanced energy demands. By contrast, FFM loss was associated with increases in GLP-1, insulin and glucagon concentrations. GLP-1 was the strongest predictor of FFM loss in the multivariate model. This finding could have significant treatment potential for the reduction of FFM loss under hypoxic conditions, which might be achieved with the help of dipeptidyl peptidase-IV (DPP-IV) inhibitors or GLP-1 analogues such as exendin-4, a pharmacological approach now commonly used for diabetes treatment. Increases in GLP-1 may represent a compensatory response to FFM loss to enhance vascular/metabolic coupling in the muscle via a synergistic effect on NO and insulin signaling pathways. Consistent with this notion, exendin-4 improved islet transplantation outcome by rescuing pancreatic islets from hypoxia-induced oxidative stress [59]. Exendin-4 has also been shown to activate Nrf2, a transcription factor that plays a central role in orchestrating cellular anti-oxidative defence and redox homeostasis, providing a potential mechanistic explanation for the anti-inflammatory effects of GLP-1 and its agonists [60]. Curiously, GLP-1 concentrations did not change in healthy, young men exposed to normobaric hypoxia for ten days [61], potentially highlighting fundamental differences between the effects of normobaric and hypobaric hypoxia on human physiology [62].

We have previously described the development of increased insulin resistance with prolonged exposure to hypobaric hypoxia [20]. However, the multivariate model reported in the present study suggests a concomitant decline of FFM and insulin concentrations during the expedition. This result is not surprising considering that, after accounting for other regulatory factors, it would be favourable in hypoxic conditions to reduce the anabolic drive and enhance tissue mobilization and energy release. In general, insulin levels tend to be increased during prolonged exposure to hypoxia [63], [64] whereas low levels of insulin have been reported in studies with short hypoxic exposure and modest altitudes [65].

### 4.6 Adipokines

Visfatin concentrations were significantly associated with FFM loss in the univariate correlation analyzes, but visfatin was not a significant predictor in the multivariate model. This adipokine is produced by adipocytes and macrophages infiltrating adipose tissue but also generated in the liver and skeletal muscle [66]. Secretion of visfatin is stimulated by inflammation and has insulin-mimetic effects such as: increased glucose uptake in adipocytes and myocytes, suppression of hepatic glucose release, and lipogenesis [67]. Ours is the first study to investigate the association between hypoxia and visfatin in humans; previous in vitro studies have shown an upregulation of visfatin gene expression in adipocytes through the activation of the HIF-1α pathway secondary to hypoxia [68], [69].

### 4.7 Strengths and limitations

The main strengths of our current study are represented by the number of subjects that participated in the eight–week expedition, the consistency of hypoxic exposure pattern and variability of response in the face of this consistent stimulus; (thereby providing an important physiological signal), the depth and richness of physiological phenotyping, and the comprehensive nature of our biomarker profiling. Another strength lies in the use of multiple biomarkers of oxidative stress to explore the relationship between hypoxia and FFM loss, which is unprecedented and uncommon for field studies. More often than not, oxidative stress markers are used interchangeably with little consideration as to their specificity, biological half-life and the nature of the oxidative insult they represent. However, our study is not without limitations. The use of BIA for the assessment of body composition at high altitude has been challenged as changes in hydration may influence the accuracy of measurements [70], [71]. However, the study used multi-frequency BIA which allows the measurement of intra- and extra-cellular compartments. Secondly, plasma osmolality during the expedition did not change, which rules out the potential interference of changes in hydration status on BIA measurements (Table S2 of the Online Supplementary Material). Skinfold thickness was also measured during the expedition to assess body composition changes, but the results obtained using this method were unreliable and have therefore not been included in this report. Finally, the generalizability of these results to the general population is limited and therefore results should be interpreted with caution. However, the primary aim of the study was to provide clues as to the effects of prolonged hypoxia on loss of FFM and identify potential mechanisms involved in the development of sarcopenia.

### 4.8 Implications for future research

Changes in GLP-1 and nitrite concentrations are significantly correlated (Fig. S3 of the Online Supplementary Material), and both are significant predictors of FFM loss. These changes are likely to represent integrated compensatory responses to increase blood flow, delivery of nutrients and overall tissue metabolic capacity. The effects of these pathways on skeletal muscle structure and performance could be tested in future animal and human studies with exposure to normoxia and hypoxia and supplementation with, for example, incretins (or DPP-IV inhibitors) and dietary nitrate. Specifically we offer candidate mechanisms that can be further evaluated in patient groups where sarcopenia is common including older patients and critically ill patients, or in those susceptible to hypoxia as a result of cardiovascular or pulmonary disease.

## 5 Conclusions

Exposure to hypobaric hypoxia resulted in a significant FFM loss that was associated with increased levels of biomarkers of the NO pathway (nitrite), markers of oxidative stress (4-HNE), inflammation (IL-6) and metabolic efficiency (GLP-1, insulin). The putative role of GLP-1 and nitrite as mediators of the effects of hypoxia on FFM is an important finding. If confirmed, nutritional and pharmacological interventions targeting GLP-1 and the Nitrite-Nitric Oxide pathways could represent promising strategies to prevent loss of skeletal muscle at high altitude and in susceptible patient groups.

## Author contributions

M.P.W.G. and M.F. are the guarantors of this work and, as such, had full access to all the data in the study and take responsibility for the integrity of the data and the accuracy of the data analysis. L.W., M.S., M.P.W.G., M.F. wrote the manuscript and researched data; H.L.R., C.A.L., K.M. researched data; H.E.M., M.G.M., M.A.S. contributed to discussion and reviewed/edited manuscript; B.O.F., M.K., D.S.M., D.Z.H.L. researched data, contributed to discussion and reviewed/edited manuscript.

## Conflict of interest statement

The authors have no conflict of interest to declare.
