# Supplementary material for: Does hypoxia play a role in the development of sarcopenia in humans? Mechanistic insights from the Caudwell Xtreme Everest Expedition
Source: Redox Biol. 2017 May 8;13:60–8. doi: 10.1016/j.redox.2017.05.004 (PMC5451185; doi:10.1016/j.redox.2017.05.004)
Supplement: Supplementary file 1 — Supplementary material [file mmc1.docx]

**ONLINE SUPPLEMENTARY MATERIAL**

| **Table S1:** Ascent profile and residence at high altitude of the team remaining at Everest Base Camp (EBC, N=10) and those climbing higher (Climbers, N=14)*. Information on altitude (m), barometric pressure (mmHg) and inspired partial pressure of oxygen (PiO_2_, mmHg) is reported for each phase. Hemoglobin concentrations (g/dl) and arterial oxygen saturation (SaO_2_, %) are reported in each group during the different phases of the expedition. | | | | | | | | |
| --- | --- | --- | --- | --- | --- | --- | --- | --- |
|  |  | **Phase/Location** | **Altitude**  **(m)** | **Barometric Pressure**  **(mmHg)** | **PiO_2_**  **(mmHg)** | **Hb**  **(g/dl)** | **SaO_2_**  **(%)** | **Plasma Samples for**  **Biomarker Assessment** |
| **Ascent to EBC** | **Day 0**  EBC lab team  Climbers | Baseline (London) | 75 | 754 | 148.0 | 13.3 (0.8)  14.2 (0.7) | 98.0 (0.8)  97.9 (1.1) | √ |
|  | **Day 1**  EBC lab team  Climbers | Kathmandu | 1300 | 650 | 126.2 | 14.2 (0.8)  14.8 (0.8) | 96.6 (1.7)  95.9 (1.3) | √ |
|  | **Day 3**  EBC lab team  Climbers | Namche | 3500 | 505 | 95.4 | 14.7 (0.7)  16.0 (1.1) | 91.0 (5.7)  90.6 (2.1) | √ |
|  | **Day 7**  EBC lab team  Climbers | Pheriche | 4250 | 461 | 86.7 | 15.0 (1.1)  15.8 (0.8) | 87.0 (4.4)  87.4 (2.3) | √ |
|  | **Day 12**  EBC lab team  Climbers | Arrival at EBC | 5300 | 404 | 74.7 | - | - | - |
| **High Altitude** | **Day 19 (Week 1)**  EBC lab team  Climbers | EBC | 5300 | 404 | 74.7 | 16.9 (1.7)  17.9 (1.5) | 77.1 (6.3)  82.6 (4.1) | √ |
|  | **Day 38 (Week 3)**  EBC lab team  Climbers | EBC  Camp 2 | 5300  6400 | 404  350 | 74.7  63.4 | 17.7 (1.1)  19.2 (2.0) | 84.1 (5.0)  75.9 (6.4) | - |
|  | **Day 56 (Week 6)**  EBC lab team  Climbers | EBC | 5300 | 404 | 74.7 | 17.3 (1.7)  18.5 (1.1) | 82.8 (5.4)  85.6 (3.9) | √ |
|  | **Day 62 (Week 7)**  EBC lab team  Climbers | EBC  Summit | 5300  8848 | 404  253 | 74.7  42.8 | - | - | - |
|  | **Day 70 (Week 8)**  EBC lab team  Climbers | EBC | 5300 | 404 | 74.7 | 17.7 (1.4)  19.4 (1.2) | 84.3 (8.1)  87.2 (3.8) | √ |

*Fourteen subjects participated in this part of the expedition of whom 8 reached the summit of Everest; 2 subjects were excluded because biomarker data were missing and therefore 12 subjects from this group were included in the final analysis. All subjects followed an identical trekking profile during their ascent to EBC and, to standardize hypoxic exposure, were prevented from excursions of more than 300 vertical metres from the group altitude at any time. For Group 1 (EBC laboratory staff), excursions were limited to within 500 vertical metres of the EBC altitude for the duration of the expedition. The climbing team (n = 14) followed an identical ascent profile until the completion of all testing at Camp 2 (6,400 m) including identical acclimatisation outings. Group 1 was not exposed to supplemental oxygen for the duration of the expedition. Group 2 was not exposed to any supplemental oxygen until the completion of testing at Camp 2. All climbers used supplemental oxygen at flow rates of 2-4 l/min for the summit climb above Camp 3 (7,100 m) and at 0.5 l/min whilst sleeping at and above Camp 3. Testing was repeated at the end of the expedition (immediately prior to departure) for all climbers at EBC (days 66 to 71).


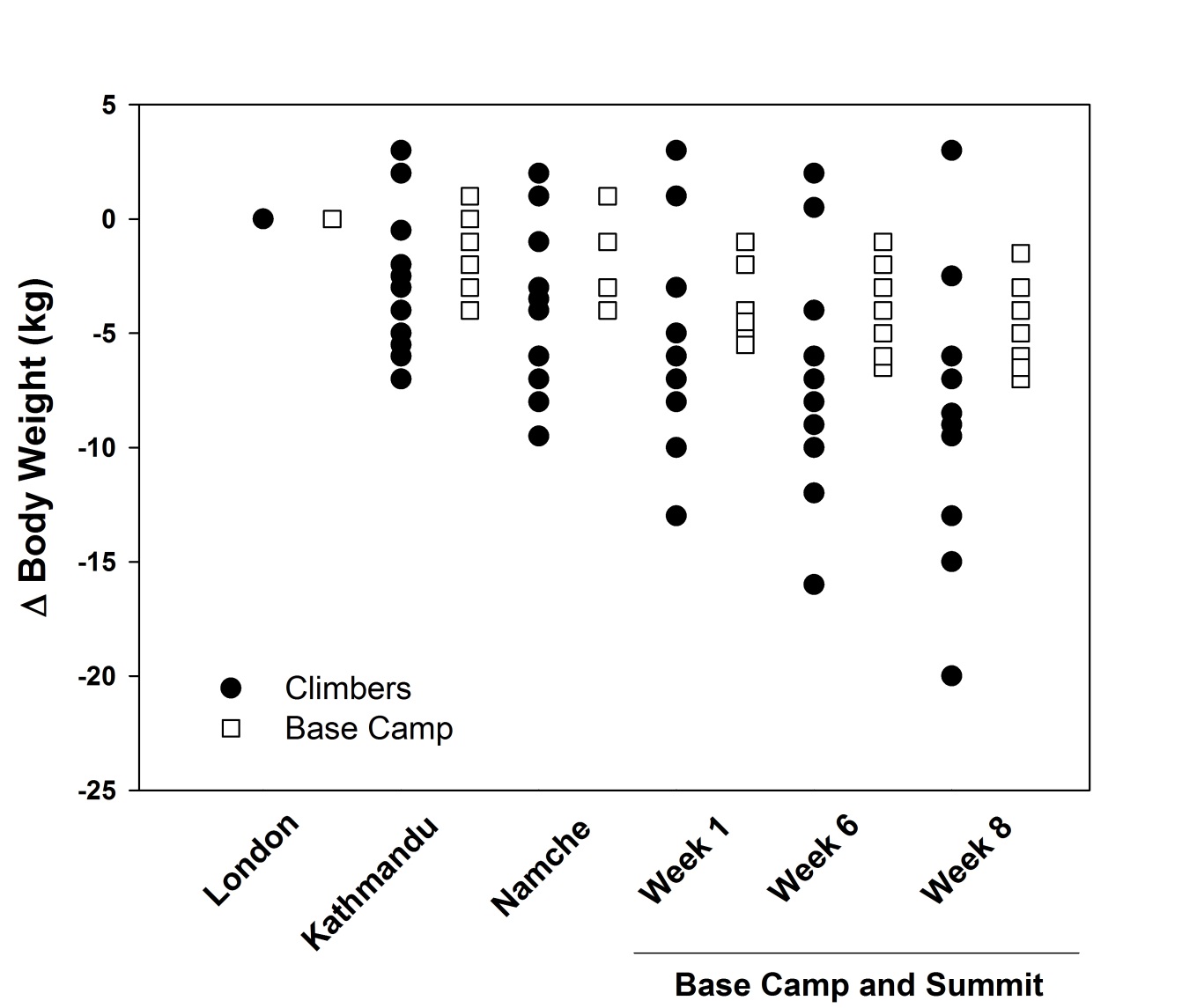


**Figure S1:** Individual changes in body weight during the expedition in climbers and base camp residents

**
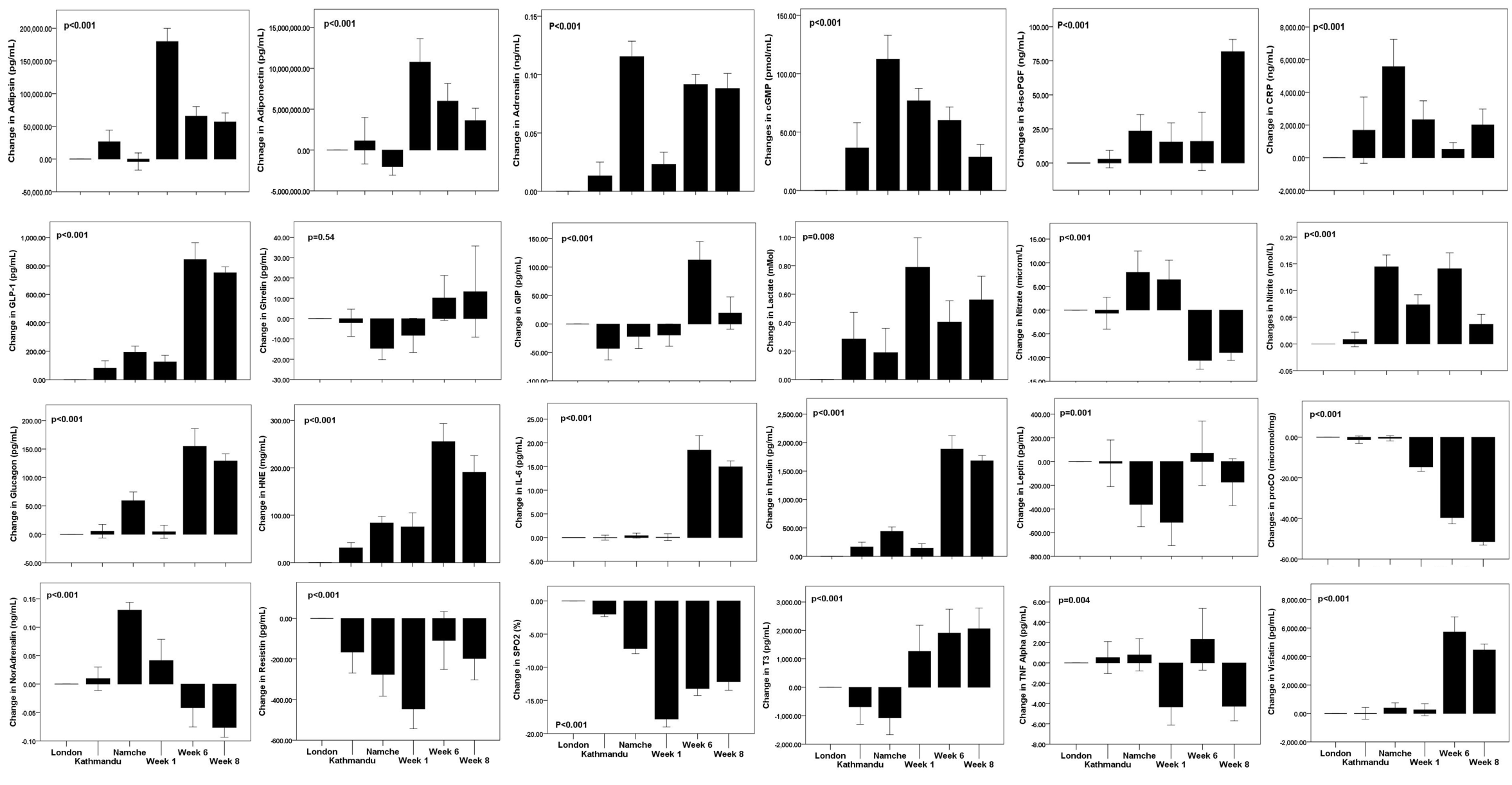
Figure S2:** Panel showing mean changes (± s.e) in metabolic biomarkers measured during the Caudwell expedition. Data were expressed relative to sea level (London). Linear mixed models for repeated measures were used to analyse whether there were significant changes in body composition during the expedition.

| **Table S2: Plasma osmolality (mOsm/kg) measured during the expedition** | | |
| --- | --- | --- |
| **Altitude** | **Mean** | **St. Dev** |
| London (75m) | 292.9 | 5.8 |
| Kathmandu (1300m) | 292.7 | 9.4 |
| Namche (3500m) | 292.7 | 9.6 |
| Pheriche (4250m) | 286.9 | 13.8 |
| Everest Base Camp Week 1 (5300m) | 289.7 | 12.3 |
| Everest Base Camp Week 6 (5300m) | 285.3 | 13.7 |
| Everest Base Camp Week 8 (5300m) | 291.1 | 12.5 |

**
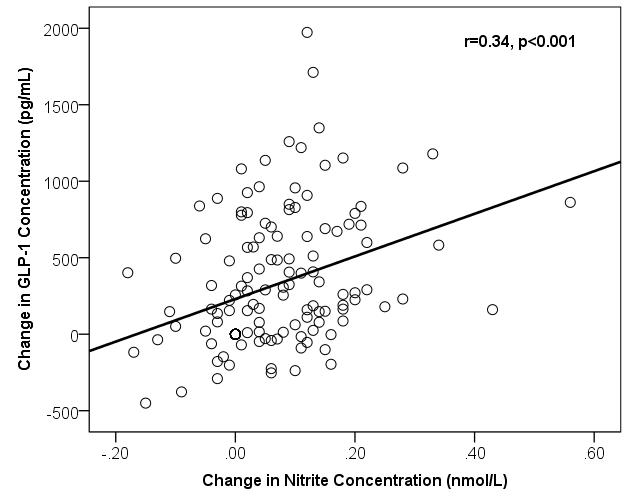
**

**Figure S3:** Correlation between changes in plasma concentrations of nitrite and GLP-1 during the expedition (N=138).
